# Common Options and Overlooked Alternative for Drainage of Inaccessible Presacral Abscess: A Case Report

**DOI:** 10.15388/Amed.2021.28.1.13

**Published:** 2021-03-15

**Authors:** Evelina Kodzis, Donatas Jocius, Ona Lapteva, Rugilė Kručaitė

**Affiliations:** Vilnius University Faculty of Medicine, M. K. Čiurlionio Str. 21, LT-03101 Vilnius, Lithuania; Vilnius University Faculty of Medicine, M. K. Čiurlionio Str. 21, LT-03101 Vilnius, Lithuania Vilnius University Hospital Santaros Klinikos, Santariškių Str.2 LT-08410 Vilnius, Lithuania; Vilnius University Hospital Santaros Klinikos, Santariškių Str.2 LT-08410 Vilnius, Lithuania; Vilnius University Faculty of Medicine, M. K. Čiurlionio Str. 21, LT-03101 Vilnius, Lithuania

**Keywords:** Case report, presacral abscess, pyogenic spondylodiscitis, transosseus drainage, transsacral approach

## Abstract

**Purpose.:**

To demonstrate options and alternative for drainage of inaccessible presacral abscess by the example of a rare clinical case of pyogenic spondylodiscitis, transsacraly drained under a combination of two interventional techniques – CT-guided bone biopsy and abscess drainage.

**Materials and methods.:**

A 55-year-old patient with history of recurrent paravertebral abscesses previously treated with antibiotic therapy was referred to our institution experiencing lower back pain and weakness in both lower extremities. Computed tomography revealed pyogenic spondylodiscitis along with left facet joint destruction and presacral abscess located in ventral sacral surface. Due to inaccessible abscess location, it was decided to perform CT-guided percutaneous transsacral abscess drainage. An 8G bone marrow biopsy needle was used to penetrate the sacrum and create a path for drainage catheter placement. Using the Seldinger technique 8 Fr drainage catheter was inserted into abscess cavity.

**Results.:**

Neither early nor late procedure-related complications occurred. Sixteen days after drainage procedure, the catheter was withdrawn as patient’s condition improved and the outflow of pus had reduced considerably.

**Conclusions.:**

Despite being rarely used, CT fluoroscopy-guided transsacral drainage approach is considered to be minimally invasive and in some cases the only viable option for drainage of pyogenic spondilodiscitis of the lumbosacral junction.

## Abstract (lt)

**Drenavimo parinktys ir alternatyvos sunkiai prieinamų presakralinių abscesų gydyme: klinikinis atvejis:**

**Tikslas.**

Supažindinti su sunkiai prieinamų dubens abscesų drenavimo parinktimis ir alternatyvomis remiantis retu klinikiniu pūlingo spondilodiscito atveju, kurį gydant buvo taikytas perkryžkaulinis drenavimas derinant dvi intervencinės radiologijos metodikas – KT kontrolėje atliekamą trepanobiopsiją ir drenavimą.

**Metodologija.**

55 metų pacientas, su žinoma ilgalaike pūlingo spondilodiscito anamneze ir anksčiau taikyta antibiotikų terapija, buvo paguldytas į ligoninę, skundžiantis apatinės nugaros dalies skausmu ir progresuojančiu silpnumu abiejose kojose. Atlikus juosmens / kryžmens srities kompiuterinės tomografijos tyrimą, buvo rastas pūlingas spondilodiscitas ir kairiojo facetinio sąnarinio tarpo destrukcija bei ventraliau kryžkaulio lokalizuotas abscesas. Atsižvelgiant į sunkiai prieinamą pūlinio lokalizaciją, nuspręsta atlikti perkryžkaulinį drenavimą kontroliuojant KT. Kryžkaulio penetracijai buvo naudojama 8G trepanobiopsinė adata, po procedūros, naudojant Seldingerio metodiką, į pūlinio ertmę įleistas 8 Fr drenavimo kateteris.

**Rezultatai.**

Po procedūros komplikacijų nebuvo. Šešioliktą parą po atliktos intervencijos drenas buvo pašalintas, nes paciento būklė gerėjo ir turinio sekrecija ženkliai sumažėjo.

**Išvados.**

Nors perkaulinis drenavimo metodas yra retai naudojamas kasdienėje praktikoje, pasaulyje jis yra pripažintas kaip mažai invazyvus ir tam tikrais atvejais vienintelis taikytinas būdas pūlingam spondilodiscitui gydyti.

**Raktažodžiai:** klinikinis atvejis, dubens abscesas, pūlingas spondilodiscitas, perkaulinis drenavimas, perkryžkaulinis drenavimas.

## Introduction

One of the most complex regions to drain abscess from is presacral region. While there are several options of drainage pathways, the most common option is a percutaneous pathway. It is considered to be safe, low-risk and effective procedure for draining pelvic abscesses which allows to avoid surgical intervention [1,2]. 

However, even having a drainage as a minimally invasive option, in some cases presacral abscesses might be difficult to access and drain. This is due to their close location to the adjacent structures, such as pelvic bones, nerves, iliac vessels, bowel, bladder and women’s reproductive organs, which often obstruct the path of a drainage catheter. Therefore, it is crucial to choose the most appropriate drainage pathway [2–5], as damaging previously named structures will lead to serious complications.

According to the literature, several alternative approaches are distinguished, including transrectal, transvaginal, transgluteal or the less common transosseous approach [3,10]. We present a case of pyogenic spondylodiscitis of the lumbosacral junction in which the only viable and safe drainage option was transsacral drainage under a combination of two interventional techniques – CT-guided bone biopsy and abscess drainage.

## Case report

### Patient

**Fig. 1.**
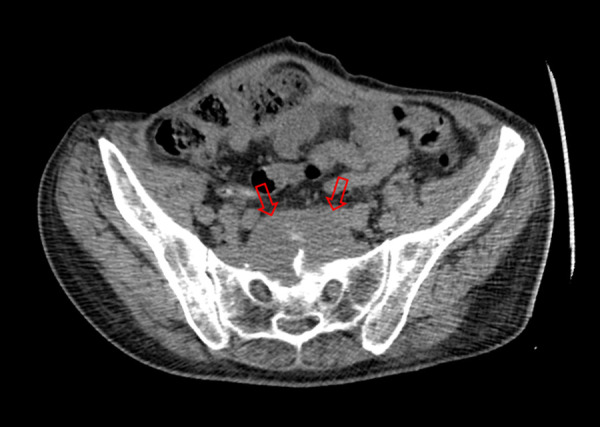
Transverse CT image showing deep pelvic abscess located at presacral region before drainage (red arrows).

A 55-year-old patient with history of drug abuse was referred to our institution experiencing lower back pain and weakness in both lower extremities. It is also known, that patient has a long history of recurrent paravertebral abscesses previously treated with antibiotic therapy. This time pain was progressive, it increased on movements and reduced on rest. Clinical examination revealed tenderness on lower back palpation, weakness on both lower extremities and infected sacral bedsore. Computed tomography (CT) was performed, and L5/S1 vertebral space pyogenic spondylodiscitis was noticed along with left facet joint destruction and presacral abscess located in ventral sacral surface (Fig. 1). 

**Fig. 2.**
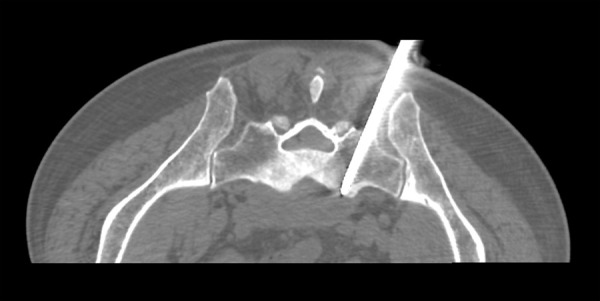
Transverse CT image showing bone marrow biopsy needle penetrating the sacrum.

Open revision, debridement and vertebral fixation were necessary, but due to infected bedsore it was decided to perform percutaneous placement of a drainage catheter instead. Because a direct anterior approach was precluded by bowel and vessels, transsacral approach was used, which was considered to be safer and more feasible. 

### Procedure

The patient was placed in a prone position and CT scan (General Electric CT VS T 64) was used to target prior to drainage. The path of the drainage catheter placement was determined in such a way that the sacral foramina, sacral canal and large vessels were avoided. After administering sufficient local anesthesia, skin incision was made, through which an 8G bone marrow biopsy needle (Medax hemax) was inserted toward the sacrum while maintaining previously set trajectory. The needle was gradually advanced through the left sacrum pedicle by applying forward pressure and rotation (Fig. 2). 

**Fig. 3.**
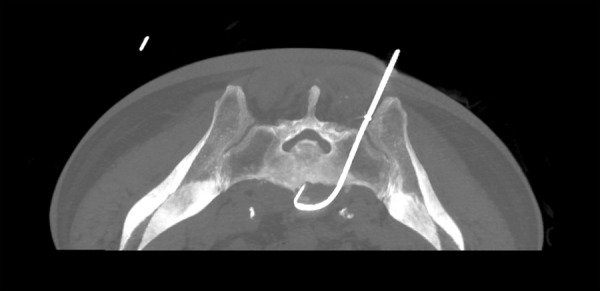
Transverse CT image showing drainage catheter placement.

After the needle had penetrated the sacrum, stylet was removed and presence of pus flow was detected. A guide wire was passed through the bone marrow biopsy needle into the abscess cavity. After confirming with CT fluoroscopy that guide wire is in the correct place, bone marrow needle was removed. A 8 Fr locking pigtail catheter (Cook medical) was passed over the guide wire into the cavity. Finally, the guide wire was removed leaving the catheter within the abscess cavity (Fig. 3).

After the catheter placement approximately 60 ml of puss was aspirated. Neither early nor late procedure-related complications like bleeding, nerve injury, bone fracture, osteomyelitis or bone abscess occurred. Sixteen days after drainage procedure, the catheter was withdrawn as patient’s condition improved, the outflow of pus had reduced considerably and inflammatory blood signs were back to normal. 

## Discussion

Taking into consideration different drainage options each approach has its own advantages and disadvantages. 

### Transgluteal approach

Transgluteal approach is considered to be the safest as its pathway minimizes the risks of peritoneal penetration, bladder injury and helps to avoid the damage of bowel and external iliac vessels. Despite being the safest, this approach requires the patient to be in the prone position which may not be optimal for several reasons. This could be difficult for patients who recently had abdominal surgery or simply for those who have colostomy bags or drainage tubes in the abdominal wall. Moreover, to lie prone could be also challenging for obese patients or for the ones suffering from respiratory diseases [6]. Sciatic nerve, sacral plexus or gluteal vessels injuries, hemorrhages and possible catheter malposition were listed as complications of transgluteal approach. Also 20% of patients who underwent this type of drainage report experiencing pain after the procedure [6,7]. 

### Transvaginal and transrectal approach

Although transgluteal approach is performed most commonly and it is considered to be the safest there are many advantages to other drainage approaches as well. Firstly, just like transgluteal, transvaginal and transrectal approaches can be easily guided by ultrasound in real-time. Secondly, especially transvaginal pathway is useful for pelvic abscesses drainage due to accessibility to most pelvic fluids. Risks of this type of procedure include bleeding and perforation of rectal or vaginal wall. It is worth mentioning that these pathways are semi-sterile, so there is a potential risk of retrograde transmission of infection [8,9]. Finally, transrectal and transvaginal routes are not suitable for long-term drainage [8].**

### Transosseus approach

Transosseus approach is useful when other approaches could not be performed. This happens when abscess is located higher than greater sciatic foramen and vessels intervene the path of the catheter. Furthermore, this approach is also suitable for obese patients when the needle path is too long due to buttock fat and overlying gluteal muscles. It is also worth using this approach when abscesses could not be accessed by other routes. It is convenient to reach the target located near the iliopsoas muscle through iliac wing. Finally, this technique allows us to avoid the penetration of peritoneum and injury to the abdominal organs minimizing the risk of infection [6]. 

There are several risks associated with the transosseus approach. These include injuring sacral nerve, gonadal vessels and ureters as well as development of osteomyelitis and pain caused by bone penetration The latter, however, could be avoided by injecting lidocaine into the periosteum before penetrating the bone [3,6]. The possibility of damaging sacral nerve could be minimized by simply having precise knowledge of pelvic anatomy. The safest location for the lumbosacral junction drainage is placing the needle through the median part of the sacrum between the sacral canal and the foramina or through the farthest lateral part of the sacrum [3] this way protecting the dural sac and nerve roots [10]. While performing the procedure CT guidance gives the operator better control of the needle and location of the surrounding structures. Despite accurate visual CT-guided control, relatively high level of skill and precise positioning of catheter is necessary in tightly restricted locations such as intervertebral space [11].

In our case transosseus approach was successfully performed, and it is considered as a rare case as there are only few of them described in scientific literature. Analogical CT fluoroscopy-guided transsacral drainage technique was performed in Tokai University Hachioji hospital [12]. In three cases of pyogenic spondylodiscitis this procedure was considered as the safest and most feasible technique. In comparison to our case there are lot of similarities including abscess localization, risk of adjacent structures damage and technique of catheter positioning. As in our case, all three drainage procedures were successful, neither early nor late procedure-related complications like bleeding, nerve injury, bone fracture, osteomyelitis or bone abscess occurred [12]. 

According to literature pyogenic spondylodiscitis or epidural abscess recurrence usually occurs within one year [13]. In our case in order to further evaluate patient’s condition extensive monitoring would be required. 

## Conclusions

Despite being rarely used, CT fluoroscopy-guided transsacral drainage approach is considered to be minimally invasive and in some cases the only viable option for drainage of pyogenic spondilodiscitis of the lumbosacral junction.
